# Emotional Labor in Knowledge-Based Service Relationships: The Roles of Self-Monitoring and Display Rule Perceptions

**DOI:** 10.3389/fpsyg.2019.00801

**Published:** 2019-04-09

**Authors:** Shenghua Huang, Hongbiao Yin, Lifang Tang

**Affiliations:** ^1^Faculty of Education, The Chinese University of Hong Kong, Shatin, Hong Kong; ^2^Faculty of Education, Northeast Normal University, Changchun, China

**Keywords:** knowledge-based service, emotional labor, well-being, self-monitoring, display rule perceptions

## Abstract

Focusing on knowledge-based service relationships, this study examined knowledge-based service workers’ (i.e., school teachers) emotional labor process and the consequential outcomes for their well-being. The study also examined the roles of two antecedents, namely, teachers’ perceptions of display rules and self-monitoring tendencies. A sample of 1,656 school teachers participated in the study. The results showed that self-monitoring generally had stronger, though maladaptive, effects than display rule perceptions on individuals’ use of emotional labor strategies (ELS) (i.e., surface acting and deep acting) and well-being (i.e., anxiety, depression, contentment, and enthusiasm). Both self-monitoring and display rule perceptions were positively related to two ELS. There were relatively stronger relationships between self-monitoring and surface acting, and between display rule perceptions and deep acting. Surface acting was positively related to anxiety and depression and negatively related to contentment and enthusiasm. Deep acting was positively related to anxiety, contentment, and enthusiasm. The examination of indirect effects showed that self-monitoring was positively related to anxiety and depression and negatively related to enthusiasm and contentment. Display rule perceptions were weakly, but positively, related to anxiety and depression. These results suggest that self-monitoring may be less beneficial than previously thought. Knowledge-based service workers’ display rule perceptions and deep acting may not necessarily be harmful to their well-being, but reflect their role identification and commitment. Theoretical contributions and practical suggestions of this study were discussed.

## Introduction

Employees’ emotional labor has emerged as an essential way to cultivate customer satisfaction and loyalty and to forge and maintain sound customer relationships (Totterdell and Holman, 2003; Côté, 2005; Grandey et al., 2005; Hülsheger et al., 2015). Emotional labor is the process by which employees display appropriate emotional expressions as required implicitly or explicitly by their organizations (Hochschild, 1983; Grandey, 2000; Brotheridge and Grandey, 2002). Unlike emotional management in a general sense, emotional labor is performed in exchange for wages (Grandey, 2000). The importance of emotional labor in service companies is self-evident. A successful service delivery depends on the positive interactions or cooperation between customers and employees (Gummesson, 1991; Gremler et al., 2001; Groth et al., 2009). Customer satisfaction and loyalty are also largely influenced by the emotional aspects of these interactions (Gremler et al., 2001; Groth et al., 2009; Hülsheger et al., 2015). Generally, previous studies have shown that friendly and authentic emotional expressions are highly predictive of customers’ positive reactions and satisfaction (Côté, 2005; Grandey et al., 2005; Hennig-Thurau et al., 2006; Groth et al., 2009). Nevertheless, the improvement of service quality through emotional labor is thought to be at the expense of employees’ well-being (Hochschild, 1983; Holman et al., 2008; Hülsheger and Schewe, 2011; Scott et al., 2012). A large number of studies have investigated the effects of the emotional labor process on employees’ mental health and well-being (Holman et al., 2008). These studies generally revealed some negative effects of emotional labor on service employees. For example, emotional display rules are believed to be an extra burden on employees (Hochschild, 1983; Brotheridge and Grandey, 2002). Emotional labor process depletes limited mental resources and leads to employees’ emotional exhaustion and reduced job satisfaction (Grandey, 2000; Hülsheger and Schewe, 2011; Humphrey et al., 2015). Positive emotional expressions achieved through faking and hiding result in employees’ sense of inauthenticity (Grandey, 2000; Humphrey et al., 2015).

Fortunately, the interests of the organization and the employees are not irreconcilable. Some researchers have suggested that emotional labor may not necessarily be harmful for employees. Emotional labor can be less stressful when employees recognize the importance of emotional labor and adopt appropriate strategies (Brotheridge and Grandey, 2002; Hülsheger and Schewe, 2011). The desired emotional expressions can be better achieved when organizations grant employees sufficient autonomy and provide them with proper instruction (Grandey, 2000). Emotional labor can even enhance employees’ sense of accomplishment and job satisfaction, especially when there is a fit between the emotional requirements of the environment and employees’ characteristics or personality (Hülsheger et al., 2013; Humphrey et al., 2015; Diefendorff et al., 2016). Despite the fact that more and more researchers tend to examine the complexity of emotional labor and its outcomes in light of different individual and organizational factors (Humphrey et al., 2015; Diefendorff et al., 2016), most studies were conducted among traditional service employees, such as bus captains, call center agents, and waiters/waitresses (e.g., Totterdell and Holman, 2003; Grandey et al., 2005; Goldberg and Grandey, 2007; Scott et al., 2012; Hülsheger et al., 2015). Few studies have investigated the emotional labor performed by high-commitment, knowledge-based service (KBS) workers, such as teachers, lawyers, and accountants.

There are three important aspects distinguishing KBS workers from traditional service employees. First, interactions between traditional service employees and customers exist in frequent, one-time, and short-term encounters, but those between KBS workers and customers involve some customized, stable, and long-term relationships (Gutek et al., 1999; Holman, 2003). Second, the emotional display rules of KBS workers are different from those of traditional service employees. As Mills and Moshavi (1999) suggested, for the high-commitment KBS workers, it is critical to exhibit both social affiliation and professional authority during their interactions with clients. These KBS workers generally need to maintain positive emotions and show friendliness in order to build relationships and elicit client cooperation. However, they still have some autonomy and can show strictness and authority when necessary (Mills and Moshavi, 1999; Holman, 2003). Third, KBS workers’ motivation to perform emotional labor could be quite different from those of traditional service employees. The primary goal of their work is to provide information and suggestions using their expertise and knowledge, rather than to ensure their clients’ comfort and relaxation. The emotional display rules for KBS workers thus are often implicit. KBS workers are less forced to perform emotional labor. The manner in which KBS workers regulate their emotions at work is seldom monitored by an immediate supervisor or colleagues. In sum, KBS workers have some but “limited” autonomy in expressing their true selves in the workplace. With different job requirements and work environments, different KBS workers (teachers, lawyers, and accountants) may interact with their clients (or students and parents) differently. Nevertheless, they still have to manage emotions and perform emotional labor at work.

The unique characteristics of KBS workers’ emotional labor make some fundamental assumptions of emotional labor questionable. For example, do KBS workers perform emotional labor for the sake of organizations and mainly in exchange for wages (Grandey, 2000)? Are employees in “people work” faced with higher emotional demands with limited personal control over emotions (Brotheridge and Grandey, 2002)? If KBS workers have more autonomy over their emotions and are not forced to display certain emotions, why are they still motivated to manage emotions at work and keep sound relationships with their clients? Thus, it is important to explore whether it is the perceived, external display rules or the internal self-monitoring tendencies that better explain KBS workers’ motivation in performing emotional labor. Display rule perceptions, on the one hand, denotes KBS workers’ awareness of the often implicit emotional rules for their interactions with clients (Brotheridge and Grandey, 2002; Diefendorff et al., 2005; Diefendorff and Greguras, 2009). Self-monitoring, on the other hand, refers to their general tendency or willingness to monitor their own emotional expressions during personal interactions (Snyder, 1974; Snyder and Gangestad, 1986; Diefendorff et al., 2005; Bono and Vey, 2007). In other words, display rule perceptions reflect the external emotional demands and rules as perceived by KBS workers, while self-monitoring represents KBS workers’ own internal monitoring tendency (Reeve et al., 2007). Previous studies on traditional service employees have examined the relationships between emotional labor and dispositional variables (e.g., self-monitoring) or situational variables (e.g., display rule perceptions) (Diefendorff et al., 2005; Bono and Vey, 2007; Holman et al., 2008). However, mixed findings are reported. These inconsistencies may be due to factors such as role identification, demand-ability fit, and contextual difference (Hülsheger et al., 2013; Humphrey et al., 2015; Diefendorff et al., 2016). Considering the uniqueness of KBS workers’ emotional labor, a closer look at the emotional labor of KBS workers can contribute to the literature. Through a comprehensive comparison between display rule perceptions and self-monitoring and their effects, we can obtain a deeper understanding of KBS workers’ emotional labor and the consequential well-being outcomes.

Thus, this study aims to examine how KBS workers’ display rule perceptions and self-monitoring are related to their different emotional labor strategies (ELS) and the consequential well/ill-being outcomes. Specifically, this study attempts to address three questions as follows: (a) whether and how are different ELS related to KBS workers’ well-being outcomes? (b) whether and how are display rule perceptions related to KBS workers’ ELS and the consequential well-being outcomes? (c) whether and how is self-monitoring related to KBS workers’ ELS and the consequential well-being outcomes? In the following sections, we review relevant theories and studies, propose and test our hypotheses, and discuss the theoretical contributions and practical implications of the results. We end by outlining research limitations and proposing directions for future studies.

## Literature Review and Hypotheses

### Knowledge-Based Service and Customer Relationships

Typically, KBS workers use their knowledge, ideas, and other intellectual capital to fulfill their work; they diagnose client priorities and recommend a course of action to their clients (Mills and Moshavi, 1999). According to Mills and Moshavi (1999), KBS workers must balance “the paradox of getting close to the client while remaining somewhat detached (p. 53).” In other words, the relationship between KBS workers and clients is both a vertical and a peer relationship. The vertical relationship secures client compliance. KBS workers need to establish their own social status and authority in the vertical relationship. They may thus carefully maintain a social and professional distance from their clients, for example, through holding objective or sometimes critical attitudes toward them (Mills and Moshavi, 1999; Gremler et al., 2001). The peer relationship, on the other hand, contributes to in-depth understanding and cooperation. That is, KBS workers need to foster psychological attachment and social affiliation with their clients (Mills and Moshavi, 1999; Gremler et al., 2001).

In a similar vein, Holman (2003) identified two types of service interaction: mass service and high commitment service. Mass service is characterized by standardized service; one-time, short-term encounters between customers and employees; and low added value (Gutek et al., 1999). High commitment service is, in contrast, characterized by customized service; long-term, relatively stable relationships between customers and employees; and high added value (Gutek et al., 1999; Holman, 2003). Workers in high commitment service industries (e.g., KBS workers) are generally better paid and have greater decision-making latitude at work. They also have more opportunities for professional development and promotion (Gutek et al., 1999; Holman, 2003).

In addition, there are no explicit emotional display rules in their organizations. As compared to traditional service workers, KBS workers are usually granted great autonomy and flexibility in maintaining both their social affiliation and professional authority (Mills and Moshavi, 1999; Holman, 2003; Diefendorff and Greguras, 2009). Professional affairs are of more relevance to their jobs. Service workers and clients constitute small and comparatively independent economic units. Their interactions are relatively private and exist both inside and outside regular organizational contexts (Gummesson, 1991; Mills and Moshavi, 1999). A direct external supervision over workers’ emotion regulation and expressions becomes less necessary or possible.

The unique characteristics of KBS workers’ emotional labor thus deserve more attention. The co-existence of formal and personal relationships between KBS workers and clients makes their emotional labor process more flexible (Gutek et al., 1999; Holman, 2003). KBS workers perform emotional labor less frequently but more intensely than employees in mass service industries (Brotheridge and Grandey, 2002). As for the emotional display rules, although positive interactions are generally desired, KBS workers sometimes are able to display neutral or even negative emotions to maintain social and professional distance from their clients (Mills and Moshavi, 1999). More importantly, KBS workers’ motivation to engage in different ELS may be quite different. Rather than being instructed or forced to perform emotional labor, KBS workers may voluntarily choose to regulate their emotions (Gosserand and Diefendorff, 2005). Specifically, KBS workers may perform emotional labor because they tend to monitor their emotional expressions during social interactions (self-monitoring), or because they identify and choose to follow the implicit emotional display rules at work (display rule perceptions) (Diefendorff et al., 2005).

School teachers, who are the focus of this study, are typical KBS workers. The agent–client relationships between teachers and students or parents are implicit and indirect. There is no contract signed directly between teachers and students. Each teacher has to act for a number of students and parents, and each student has several teachers who help them make decisions about their learning. Nevertheless, there are “invisible” contracts between teachers and students. On the one hand, most teachers understand their responsibilities and try to act in the best interests of their students. On the other hand, most students and parents trust teachers and let teachers decide on the proper courses of their learning and school activities. Actually, the co-existence of peer and vertical relationships, which is the most important characteristic of Mills and Moshavi’s (1999) KBS workers, is much more commonly observed among teacher-student relationships than other agent-client relationships.

As KBS workers, teachers use their content knowledge and teaching experience to teach students, help students to find best ways to learn, and cooperate with colleagues and parents to fulfill their work. There are relative stable and long-term relationships between the teacher and the students. A teacher not only need to care for and about the students, but also establish authority and maintain classroom rule and discipline. In addition, there are no slogans such as “the students/parents are always right” for a teacher. Different teachers may have totally different understanding of the emotional display rules at the school (Hargreaves, 2000; Newberry, 2010; Yin, 2016). For example, some teachers may believe that teaching should be professional and emotion-neutral; others may believe that positive emotional interactions between teachers and students contribute to student learning and performance; and still others may believe that caring and loving their students are the most important responsibilities on their part. These dynamics among teachers reflect the unique nature of KBS workers’ emotional labor. A further investigation on the relationships among teachers’ display rule perceptions, self-monitoring, emotional labor and the consequential well-being outcomes will contribute to an in-depth understanding of the complexity of KBS workers’ emotional labor.

### Emotional Labor Strategies and Well/Ill-Being

Emotional labor means managing one’s emotions to earn a wage (Hochschild, 1983). When performing emotional labor, employees and workers have to use appropriate strategies to regulate their inner feelings or external expressions. Two essential types of ELS are surface acting and deep acting. Surface acting involves hiding or faking emotions and leads to a consequent dissonance between expressed emotions and inner feelings. Deep acting refers to modifying or changing one’s inner feelings and thus leads to the natural expressing of emotions required (Hochschild, 1983; Grandey, 2000; Hülsheger and Schewe, 2011). According to Grandey (2000). For example, when getting irritated by a student, a teacher can remain calm and positive through hiding his or her true emotions (i.e., surface acting). The teacher may also reappraise the situations and attribute students’ misbehaviors to a lack of self-control or disadvantaged family backgrounds (i.e., deep acting), and thus feel more compassion and less anger. Additionally, rather than to regulate the emotions directly, a teacher may also choose to leave the classroom or suspend the on-going classroom activities to cope with these situations. The initiation of the emotion regulation process is closely related to situational cues, such as general interaction expectations, display rules, and real-time positive or negative affective events (Gosserand and Diefendorff, 2005; Goldberg and Grandey, 2007). The strategies adopted in the emotion regulation process and their effects vary across different individual characteristics (e.g., gender, affectivity, emotional self-monitoring, and emotional intelligence) and organizational factors (i.e., job autonomy, social supports) (Grandey, 2000; Totterdell and Holman, 2003; Hülsheger et al., 2013). The empirical results suggest that individuals with higher emotional capacities (e.g., emotional intelligence, emotional knowledge) are found to use more deep acting than surface acting (Brotheridge and Grandey, 2002; Yin et al., 2017). Perceived emotional job demands are found to increase the use of both types of ELS. Job autonomy and social support, on the other hand, can provide a buffer against the negative effects of emotional labor (Totterdell and Holman, 2003; Diefendorff et al., 2005; Holman et al., 2008).

In general, surface acting is more maladaptive than deep acting, though deep acting can also do harm to individuals’ well-being (Côté, 2005; Holman et al., 2008; Hülsheger and Schewe, 2011). Previous theories and research generally suggest that surface acting is negatively related to individuals’ well-being, because the process leads to mental resource depletion, a sense of inauthenticity, and negative feedback from customers (Grandey, 2000; Côté, 2005; Hülsheger and Schewe, 2011; Hülsheger et al., 2013; Humphrey et al., 2015). However, the relationship between deep acting and well-being indicators is less consistent: deep acting is thought to have a dual effect on individuals’ well-being (Hülsheger and Schewe, 2011). Although deep acting depletes mental resources and leads to emotional exhaustion, it is also related to positive customer feedback and a sense of pride and accomplishment (Grandey, 2000; Hülsheger and Schewe, 2011; Humphrey et al., 2015). Consistently, empirical studies have found that surface acting is positively related to emotional exhaustion, depersonalization, and a lack of personal accomplishment. Surface acting is also found to be negatively related to job satisfaction and self-efficacy (Goldberg and Grandey, 2007; Hülsheger and Schewe, 2011; Yin, 2016). Deep acting is generally found to be positively related to emotional exhaustion. However, mixed findings were reported in terms of the relationships between deep acting and job satisfaction and personal accomplishment (Hülsheger and Schewe, 2011; Yin, 2016).

For teachers or KBS workers in general, the emotional labor process is more flexible. However, there should be similar underlying mechanisms, through which ELS influence their well/ill-being outcomes. That is, when surface acting is adopted, a teacher has to spend sustained effort and mental resources (Brotheridge and Grandey, 2002). The feelings of fatigue and inauthenticity can be reflected in their frequent experience of uneasiness and depression as well as a lack of enthusiasm or calmness during work (Grandey et al., 2005). When deep acting is adopted, the mental resources depletion process also leads to the unpleasant experience of anxiety or depression (Hülsheger and Schewe, 2011). However, the faithful effort of teachers may also contribute to more positive attitudes and feelings toward their work (e.g., contentment, enthusiasm) (Brotheridge and Grandey, 2002; Totterdell and Holman, 2003; Yin et al., 2017). Given this dual process, it is necessary to explore the effects of ELS on ill-being and well-being indicators, respectively. In other words, ill-being and well-being should be treated as two distinct criteria rather than as two opposite ends of the continuum of well-being. Thus, based on Warr’s (1990) conception of the four dimensions of well-being, which comprise two well-being indicators (contentment, enthusiasm) and two ill-being indicators (anxiety, depression), the following relationships are hypothesized:

*H1a*: *Surface acting is positively related to anxiety and depression, and negatively related to contentment and enthusiasm.*
*H1b*: *Deep acting is positively related to anxiety and depression, and positively related to contentment and enthusiasm.*

### Display Rule Perceptions

Emotional display rules specify the appropriate kinds of emotional expressions and the ways emotions should be displayed (Brotheridge and Grandey, 2002; Diefendorff et al., 2005; Goldberg and Grandey, 2007). A widely adopted classification of display rule perceptions distinguishes between positive display rule perceptions (i.e., rules for expressing positive emotions) and negative display rule perceptions (i.e., rules for suppressing negative emotions) (Brotheridge and Grandey, 2002; Diefendorff et al., 2005). Although it is theoretically plausible that being required to “express positive emotions” is more palatable than to “hide negative emotions,” many researchers have failed to differentiate the two dimensions of positive and negative perceptions (Gosserand and Diefendorff, 2005; Goldberg and Grandey, 2007). In fact, displaying positive emotions inevitably means not displaying negative ones, and vice versa. Therefore, it is simplest to classify display rules into three types: to express positive emotions (and thus hide negative ones), to express negative emotions (and thus hide positive ones), and to remain neutral (Côté, 2005). In the present study, we thus treat display rule perceptions as a single-dimension variable, which integrates expressing positive emotions and hiding negative ones (Gosserand and Diefendorff, 2005).

Since emotional display rules may be set explicitly or implicitly by organizations, the subjective perceptions of display rules can vary across individuals, especially when it comes to implicit rules (Diefendorff et al., 2005; Diefendorff and Greguras, 2009). Allen et al. (2014) suggested that people in collectivistic cultures are more concerned with social harmony and group cooperation, and thus perceive a greater number of emotional display rules and report more frequent use of ELS. For KBS workers, individualized perceptions of display rules are more common. As discussed above, the emotional display rules for KBS workers are generally implicit. Teachers are generally required to express positive emotions toward students and parents, although some negative emotions of teachers are still acceptable at school (Hargreaves, 2000). However, teachers may have different understandings of their expected roles and the appropriate emotional expressions at work. The lack of direct external supervision also makes it difficult to ensure each teacher has the same perception of and responses toward these emotional display rules.

As stated earlier, situational cues, such as emotional job demands, display rules, and real-time positive or negative affective events, may make emotional labor necessary (Grandey, 2000; Totterdell and Holman, 2003). Empirically, Brotheridge and Grandey (2002) found that both positive and negative display rule perceptions were positively related to emotional labor. Previous findings also indicated that the use of both types of emotional labor (i.e., surface acting and deep acting) increased in accordance with employees’ awareness of emotional display rules/demands (Gosserand and Diefendorff, 2005; Goldberg and Grandey, 2007). For teachers, it is likely that highly perceived display rules will lead teachers to performing more emotional labor. Thus, consistent with previous studies, we suppose that display rule perceptions will be positively related to both surface acting and deep acting, depending on teachers’ predispositions (Brotheridge and Grandey, 2002; Diefendorff et al., 2005). However, it should be noted that, considering the implicit display rules and limited external supervision, teachers are not forced to hide or fake their emotions even with high display rule perceptions. Thus, the high display rule perceptions of teachers may tend to encourage them to use more deep-level solutions, rather than force themselves to hide or fake emotions. In other words, the positive relationship between display rule perceptions and deep acting should be stronger than that between display rule perceptions and surface acting. We thus hypothesize as follows:

*H2a*: *Display rule perceptions are positively related to both surface acting and deep acting; there is a stronger relationship between display rule perceptions and deep acting.*

Additionally, since surface acting and deep acting are predictive of teacher well/ill-being, there should be indirect effects of display rule perceptions on teachers’ well/ill-being outcomes via ELS. Researchers suggested that the emotional labor process (especially surface acting) initiated by display rules depletes attentional resources and energy (Goldberg and Grandey, 2007). Display rules and an inefficient emotional labor process may induce emotion-rule dissonance. That form of person-role conflict is further predictive of emotional exhaustion and job dissatisfaction (Holman et al., 2008; Hülsheger and Schewe, 2011). However, if display rule perceptions lead to the use of more adaptive ELS (i.e., deep acting in the case of teachers), these unfavorable effects should be less severe. Combining previous assumptions, we thus hypothesize that the perceived display rules and extra effort in emotional labor are stressful and may lead to anxiety and depression. However, teachers’ faithful effort enhanced by a high display rule perceptions may balance the negative effect of surface acting, and thus generally contribute to more positive attitudes and feelings (e.g., contentment, enthusiasm). The following relationships are hypothesized:

*H2b*: *Surface acting and deep acting mediate the relationship between display rule perceptions and well/ill-being indicators.*
*H2c*: *The total indirect effects of display rule perceptions on anxiety and depression are positive, while those on contentment and enthusiasm are also positive.*

### Self-Monitoring

According to Snyder (1974), self-monitoring of expressive behavior refers to individuals’ tendency to control and regulate their expression according to situational cues. Low self-monitors are more likely to be themselves and exhibit consistency between inner feelings and external expressions (Day et al., 2002; Scott et al., 2012). By contrast, high self-monitors tend to hide their inner feelings and attune their facial expressions, voice, and posture to situational cues (Bono and Vey, 2007; Scott et al., 2012). Moreover, an individual’s tendency to control and regulate his or her expressions may vary across day-to-day situations. For example, the need for self-monitoring decreases if individuals are interacting with close friends/relatives under familiar circumstances (Hülsheger et al., 2013; Huang et al., 2018).

Self-monitoring has long been treated as a desirable quality and associated with better personal performance, such as desirable public appearance, better impression management, harmonious relationships, and leadership (Snyder and Gangestad, 1986; Day et al., 2002).

However, the relationships between self-monitoring and ELS and the consequential well-being outcomes remain controversial. Some studies have suggested that self-monitoring is negatively related to individuals’ well-being. In a meta-analytic review of self-monitoring, negative relationships were found between self-monitoring and organizational commitment and job satisfaction, while positive relationships were found between self-monitoring and role conflict and role ambiguity (Day et al., 2002). In a similar vein, Baumeister et al.’s (1998) research on ego depletion also suggested negative effects of self-monitoring. According to Baumeister et al. (1998), self-regulation and other forms of volition lead to expenditure of limited resources, which in turn contributes to burnout and ill-health. In this respect, self-monitoring is thought to be related to maladaptive coping and ill-being (Diefendorff et al., 2005).

Another group of studies, however, believed that high self-monitors were more skilled and capable of performing emotional labor than low self-monitors. For example, high self-monitors are thought to have acute awareness of situational cues and show more willingness and efficiency in regulating emotional expressions (Bono and Vey, 2007; Scott et al., 2012). Some studies have also suggested a buffering effect of self-monitoring on the maladaptive relationship between surface acting and job satisfaction/work withdrawal (Scott et al., 2012). This second line of studies seems to conceptualize self-monitoring as inherently related to better emotion regulation skills and capacities, rather than as a willingness or tendency to monitor emotional expressions. Nevertheless, based on a social interaction model, Côté (2005) suggested that emotion regulation can increase work strain when receivers (i.e., customers) give negative responses, or decrease work strain if they give positive responses. Thus, assuming high self-monitors are more sensitive to situational cues (customer responses in this case), both the positive and the negative relationships between emotion regulation and work strain shall be stronger for high self-monitors (Côté, 2005).

Unlike display rule perceptions which reflect the external emotional demands and rules as perceived by KBS workers, KBS workers’ self-monitoring reflects an individual tendency in terms of emotional management (Reeve et al., 2007). Thus, it is not expected that KBS workers’ self-monitoring would have any context-specific characteristics different from those of traditional service employees. In addition, compared to display rule perceptions, KBS workers’ internalized, self-determined monitoring of their emotional expressions is more predictive of their actions and behaviors (Reeve et al., 2007). The evidence regarding the negative effects of self-monitoring on individual well-being reveals that self-monitoring may not be as adaptive as some researchers suggest. Rather, as a tendency to regulate expressive behavior or as a dimension of self-regulation, self-monitoring and the consequent emotional labor process deplete individuals’ finite pool of resources, induce role conflict, and are related to work strain and reduced well-being (Baumeister et al., 1998; Brotheridge and Grandey, 2002; Day et al., 2002; Holman et al., 2008). In other words, high self-monitoring may be more predictive of emotional labor frequency but not effectiveness. While a high level of self-monitoring is predictive of both types of ELS, the relative strength of these relationships may depend on other factors, such as the availability of ego resources or individuals’ emotional capacities (e.g., emotional intelligence, emotional knowledge) (Baumeister et al., 1998; Grandey, 2000). Thus, the following relationships are hypothesized:

*H3a*: *Self-monitoring is positively related to both surface acting and deep acting.*
*H3b*: *Surface acting and deep acting mediate the relationship between self-monitoring and well/ill-being indicators.*
*H3c*: *The total indirect effects of self-monitoring on anxiety and depression are positive, while those on contentment and enthusiasm are negative.*

**FIGURE 1:**
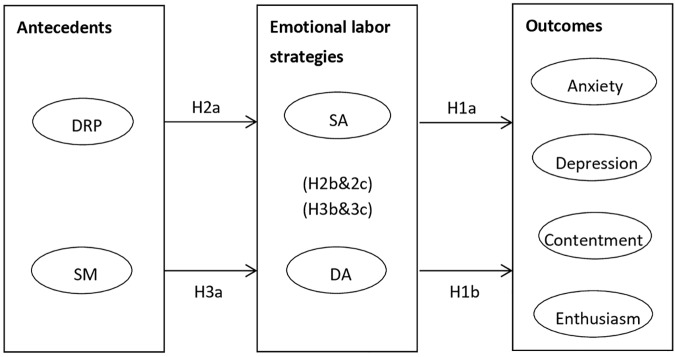
The hypothesized model. DRP, Display rule perceptions; SM, Self-monitoring; SA, surface acting; DA, deep acting; H2b&3b and H2c&3c denote the mediating roles of emotional labor strategies as well as the indirect effects of antecedents on outcomes respectively.

The hypothesized model is presented in Figure 1.

## Materials and Methods

### Procedures and Participants

Consistent with institutional review board procedures, this study was carried out in accordance with the recommendations of Survey and Behavioral Research Ethics Committee at the Chinese University of Hong Kong with written informed consent from all subjects. All subjects gave written informed consent in accordance with the Declaration of Helsinki. The protocol was approved by the Survey and Behavioral Research Ethics Committee.

The participants in this study were a sample of school teachers in Hong Kong. We connected with school principals and invited teachers to participate in the survey. Teachers had the full autonomy to decide whether to participate in the survey or not. All completed questionnaires were mailed by the participants directly to the authors. Anonymity was guaranteed. The final sample contained a total of 1,656 teachers from 38 primary schools (n1 = 1,115) and 16 secondary schools (n2 = 541). The valid response rate was 41.4%. The sample comprised 465 male participants (28.1%), 1,167 female participants (70.5 %), and 24 participants who did not report their gender (1.4%). In addition, 68 participants (4.1%) reported that they were principals in their schools, 630 (38.1%) were subject panel heads, 889 (53.7%) were regular teachers, and 69 (4.2%) did not specify their positions. In terms of their educational backgrounds, most reported a bachelor’s (680 participants, 41.1%) or master’s degree (928 participants, 56.0%). Table 1 summarizes these demographic factors in detail.

**Table 1:** Participants’ demographics.

	Category	Frequency	Percentage (%)
School type	Primary school	1115	67.3
	Secondary school	541	32.7
Gender	Male	465	28.1
	Female	1167	70.5
	Others	24	1.4
Position	Principals	68	4.1
	Panel heads	630	38.0
	Ordinary teachers	889	53.7
	Others	69	4.2
Education	Doctoral degree	5	0.3
	Master’s degree	928	56
	Bachelor’s degree	680	41.1
	Junior college diploma	32	1.9
	Others	11	0.7
Years of experience	1–3 years	227	13.7
	4–10 years	346	20.9
	11–20 years	620	37.4
	21–30 years	355	21.4
	31 years and above	101	6.1
	Others	7	0.5
Total		1656	100

### Instruments

A total of 34 items were used to operationalize the eight latent variables. Participants rated each item on a five-point Likert scale from “1 = strongly disagree (or never)” to “5 = strongly agree (or always)” according to the extent to which they agreed with the statements or the frequency of certain experiences. Translation and back-translation procedures were strictly followed to convert the English scales into Chinese versions.

#### Display Rule Perceptions

Teachers’ perceptions of display rules were measured using a five-item scale adapted from Diefendorff et al. (2005). While the original inventory measures two dimensions of display rule perceptions (i.e., positive and negative display rule perceptions), our data did not support a two-dimension structure. The final scale used in this study measures an integrated perception of the extent to which participants were required to display positive emotions while hiding negative ones (Gosserand and Diefendorff, 2005; Goldberg and Grandey, 2007). Sample items include “I am expected to suppress my bad moods or negative reactions to students” and “My school expects me to try to act excited and enthusiastic in my interactions with students.” The Cronbach’s α coefficient of the scale obtained in this study was 0.79.

#### Self-Monitoring

A short version of Snyder and Gangestad’s (1986) Self-monitoring Scale was used to measure participants’ self-monitoring tendencies. Consistent with the practice of previous studies, the short version only includes seven non-reversely coded items from the original version (Scott et al., 2012). The participants were asked to report whether they were flexible and often attuned their public appearances to situational cues. Sample items include “In different situations and with different people, I often act like very different persons” and “I’m not always the person I appear to be.” The Cronbach’s *α* coefficient of self-monitoring obtained in this study was 0.72.

#### Emotional Labor Strategies

Surface acting and deep acting were measured using the ELS scale. The scale was adapted and validated by Diefendorff et al. (2005) basing on the previous works of Kruml and Geddes (2000) and Grandey (2003). Surface acting was measured by six items including “I fake the emotions I show when dealing with students or their parents,” and deep acting was measured by four items including “I try to actually experience the emotions that I must show to students or their parents.” The Cronbach’s α coefficients of surface acting and deep acting obtained in this study were 0.90 and 0.74, respectively.

#### Well/Ill-Being Indicators

Warr’s (1990) 12-item scale consisting of both ill-being and well-being indicators was used to measure the consequential criteria. The participants were asked to report the frequency with which they experienced the following four sets of feelings over the previous few weeks: tension, uneasiness, and worry (anxiety); depression, gloom, and misery (depression); calm, contentment, and relaxation (contentment); and cheerfulness, enthusiasm, and optimism (enthusiasm). The Cronbach’s α coefficients of anxiety, depression, contentment, and enthusiasm obtained in this study were 0.91, 0.89, 0.81, and 0.88, respectively.

### Analyses

Two main statistical procedures, confirmatory factor analysis (CFA) and structural equation modeling (SEM), were conducted using Mplus 7. SEM was used to test the hypothesized model because it allows a set of relationships between multiple independent and dependent variables. Simultaneously, the latent construct also adjusts for any measurement error in both dependent and independent variables (Schreiber et al., 2006). Basic statistical analyses were conducted using SPSS 20. Chi-square values (χ^2^), degree of freedom (*df*), and the corresponding significant values (*p*), in addition to other model fit information such as the root mean square error of approximation (RMSEA), the standardized root mean square residual (SRMR), the comparative fit index (CFI), and the Tucker-Lewis Index (TLI), were reported. Model fit is excellent when the CFI and TLI are greater than 0.95 and acceptable when the NNFI and CFI are no less than 0.90. In addition, RMSEA and SRMR must be less than 0.06 and 0.08 for an excellent model fit, and 0.08 and 0.10 for an acceptable model fit (Schreiber et al., 2006). To test the indirect effects, bootstrapping technique (bootstrap samples = 2,000) was adopted, and the 95% confidence intervals (95% CIs) were calculated (Preacher and Hayes, 2008).

## Results

### Reliability, Descriptive Statistics, and Correlations

Table 2 reports the latent correlations among variables investigated in the present study and their respective mean values (ranging from 2.59 to 3.53), standard deviations (ranging from 0.55 to 0.95), and Cronbach’s α coefficients (ranging from 0.72 to 0.90). In average, the teachers reported highest values for display rule perceptions (*M* = 3.53) and the lowest values for their experiences of depression (*M* = 2.59). The Cronbach’s *α* coefficients were all greater than the generally accepted criterion (α > 0.70). In addition, the factor loadings of each item were all significant, ranging from 0.32 to 0.91. These latent variables had small to medium correlations with each other, except for the high correlations between anxiety and depression (*r* = 0.86) and between contentment and enthusiasm (*r* = 0.91). No significant correlations were found between deep acting and either contentment or enthusiasm, nor between self-monitoring and enthusiasm.

**Table 2:** Descriptive statistics and latent factor correlations based on CFA.

	1	2	3	4	5	6	7	8
(1) Display rule perceptions	–							
(2) Self-monitoring	0.33^∗∗^	–						
(3) Surface acting	0.30^∗∗^	0.59^∗∗^	–					
(4) Deep acting	0.38^∗∗^	0.33^∗∗^	0.38^∗∗^	–				
(5) Anxiety	0.24^∗∗^	0.24^∗∗^	0.42^∗∗^	0.24^∗∗^	–			
(6) Depression	0.23^∗∗^	0.23^∗∗^	0.42^∗∗^	0.20^∗∗^	0.86^∗∗^	–		
(7) Contentment	−0.13^∗∗^	−0.09^∗∗^	−0.39^∗∗^	−0.06	−0.62^∗∗^	−0.64^∗∗^	–	
(8) Enthusiasm	−0.08^∗∗^	−0.03	−0.33^∗∗^	−0.04	−0.50^∗∗^	−0.57^∗∗^	0.91^∗∗^	–
Mean	3.53	2.98	2.89	3.46	2.98	2.59	2.89	3.06
*SD*	0.55	0.56	0.79	0.56	0.9	0.95	0.75	0.78
Cronbach’s α	0.79	0.72	0.9	0.74	0.91	0.89	0.81	0.88

*^∗∗^p < 0.01; SD, standard deviation. Goodness-of-fit index: χ^2^ = 2978.35, df = 499, p = 0.00, RMSEA = 0.055, SRMR = 0.057, CFI = 0.92, TLI = 0.90.*

An eight-factor model was tested using CFA. The eight factors were display rule perceptions, self-monitoring, surface acting, deep acting, enthusiasm, contentment, anxiety, and depression, respectively. Each item was loaded on its corresponding factors. Factors were correlated with each other. The results of CFA generally confirmed the construct validity of the measurement with an acceptable model fit (χ^2^ = 2978.35, *df* = 499, *p* = 0.00, RMSEA = 0.055, SRMR = 0.057, CFI = 0.92, TLI = 0.90).

### SEM Results

**FIGURE 2:**
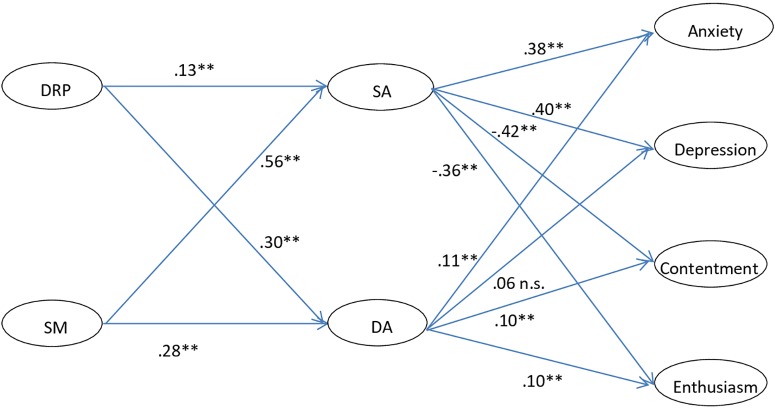
The SEM results of the hypothesized model (*n* = 1656). DRP, Display rules perception; SM, Self-monitoring; SA, Surface acting; DA, Deep Acting; n.s., not significant, ^∗∗^*p* < 0.01; Goodness-of-fit index: χ^2^ = 3079.59, *df* = 508, *p* = 0.00, RMSEA = 0.055, SRMR = 0.063, CFI = 0.91, TLI = 0.90.

The results of SEM are presented in Figure 2. The results indicate an acceptable model fit of the hypothesized model: χ^2^ = 3079.59, *df* = 508, *p* = 0.00, RMSEA = 0.055, SRMR = 0.063, CFI = 0.91, TLI = 0.90. As shown in Figure 2, all four well/ill-being indicators had stronger relationships with surface acting than with deep acting. Surface acting was positively associated with anxiety (β = 0.38, *p* < 0.01) and depression (β = 0.40, *p* < 0.01), and negatively associated with contentment (β = −0.42, *p* < 0.01) and enthusiasm (β = −0.36, *p* < 0.01) (H1a supported). Deep acting was positively associated with anxiety (β = 0.11, *p* < 0.01), contentment (β = 0.10, *p* < 0.01), and enthusiasm (β = 0.10, *p* < 0.01), although no significant relationship was found between deep acting and depression (β = 0.06, *n*.*s*.). Thus, H1b was generally supported, except for the non-significant relationship between deep acting and depression. Furthermore, display rule perceptions and self-monitoring were both positively related to the two types of ELS (H2a and H3a supported), with self-monitoring having a stronger relationship with both types of strategy. In addition, the relationship between display rule perceptions and deep acting (β = 0.30, *p* < 0.01) was stronger than that between display rule perceptions and surface acting (β = 0.13, *p* < 0.01), while the relationship between self-monitoring and deep acting (β = 0.28, *p* < 0.01) was weaker than that between self-monitoring and surface acting (β = 0.56, *p* < 0.01).

### Indirect Effects

Table 3 shows the indirect effects of display rule perceptions and self-monitoring on the four well/ill-being indicators via two types of ELS. A 95% CI including zero indicates a non-significant indirect effect, and a 95% CI not including zero indicates a significant indirect effect. In general, the results of the indirect effects via ELS indicated that deep and surface acting mediated the relationships between display rule perceptions or self-monitoring and well/ill-being indicators, generally supporting H2b and H3b, although there were no significant indirect effects on depression via deep acting.

**Table 3:** The estimates of indirect effects and the 95% confidence intervals.

Indirect effects		Estimate	95% CIs	
			Lower 2.5%	Upper 2.5%
**Display rule perceptions**				
DRP-Anxiety		0.08	0.05	0.12
	via SA	0.05	0.02	0.08
	via DA	0.03	0.01	0.05
DRP-Depression		0.07	0.03	0.10
	via SA	0.05	0.02	0.08
	via DA	0.02	0.00	0.03
DRP-Contentment		−0.02	−0.06	0.01
	via SA	−0.05	−0.08	−0.02
	via DA	0.03	0.01	0.05
DRP-Enthusiasm		−0.02	−0.05	0.01
	via SA	−0.05	−0.07	−0.02
	via DA	0.03	0.01	0.05
**Self-monitoring**				
SM-Anxiety		0.25	0.18	0.31
	via SA	0.22	0.15	0.28
	via DA	0.03	0.01	0.05
SM-Depression		0.24	0.17	0.31
	via SA	0.23	0.16	0.29
	via DA	0.02	0.00	0.03
SM-Contentment		−0.21	−0.28	−0.14
	via SA	−0.24	−0.31	−0.16
	via DA	0.03	0.01	0.05
SM-Enthusiasm		−0.17	−0.23	−0.11
	via SA	−0.20	−0.26	−0.14
	via DA	0.03	0.01	0.05

*CIs, Confidence Intervals; DRP, Display rule perceptions; SM, Self-monitoring; SA, surface acting; DA, deep acting.*

In further investigating the total indirect effects of display rule perceptions and self-monitoring, it was found that self-monitoring had stronger total indirect effects compared to display rule perceptions on the four well/ill-being indicators. There were significant and positive, though weak, total indirect effects of display rule perceptions on anxiety (Estimate = 0.08, 95% CI = [0.05,0.12]) and depression (Estimate = 0.07, 95% CI = [0.03,0.10]). No significant indirect effects were found between display rule perceptions and contentment (Estimate = −0.02, 95% CI = [−0.06,0.01]) or enthusiasm (Estimate = −0.02, 95% CI = [−0.05,0.01]). In other words, the negative effects on contentment and enthusiasm via surface acting and positive effects via deep acting balanced each other out, and resulted in non-significant indirect effects between display rule perception and contentment or enthusiasm. Thus, a positive total indirect relationship between display rule perception and ill-being was supported, but a positive indirect relationship between display rule perception and well-being was rejected (H2c).

In contrast, both the total positive indirect effect of self-monitoring on anxiety (Estimate = 0.25, 95% CI = [0.18,0.31]) and depression (Estimate = 0.24, 95% CI = [0.17,0.31]) and its total negative indirect effect on contentment (Estimate = −0.21, 95% CI = [−0.28,−0.14]) and enthusiasm (Estimate = −0.17, 95% CI = [−0.23,−0.11]) were significant, which supported H3c.

Table 4 summarizes the results of the hypothesis tests.

**Table 4:** A summary of the hypotheses and results.

	Hypotheses	Results	Remarks
H1a	Positive relationships between SA and A and D	Supported	
	Negative relationships between SA and C and E	Supported	
H1b	Positive relationships between DA and A and D	Partially supported	A non-significant DA-D relationship, further suggesting an acceptable “maladaptive” effect of DA;
	Positive relationships between DA and C and E	Supported	Highlighting the adaptive role of DA
H2a	Positive relationships between DRP and SA and DA	Supported	A stronger relationship found between DPR and DA
H2b	SA as mediators between DRP and A, D, C, and E	Supported	
	DA as mediators between DRP and A, D, C, and E	Partially supported	A non-significant indirect effect of DRP on D via DA
H2c	Positive indirect relationships between DRP and A and D	Supported	Significant, though **weak**, total indirect effects;
	Positive indirect relationships between DRP and C and E	**Rejected**	Non-significant total indirect effects
H3a	Positive relationships between SM and SA and DA	Supported	A stronger relationship found between SM and SA
H3b	SA as mediators between SM and A, D, C, and E	Supported	
	DA as mediators between SM and A, D, C, and E	Partially supported	A non-significant indirect effect of SM on D via DA
H3c	Positive indirect relationships between SM and A and D	Supported	
	Negative indirect relationships between SM and C and E	Supported	

*DRP, Display rule perceptions; SM, Self-monitoring; SA, surface acting; DA, deep acting; A, Anxiety; D, Depression; C, Contentment; E, Enthusiasm.*

## Discussion

Focusing on the emotional labor of KBS workers, this study has summarized several unique characteristics of KBS relationships and identified two essential variables that impact KBS workers’ strategy choices and consequential well/ill-being indicators. The results show that the two essential variables had similar impacting patterns, though with different strengths. In general, self-monitoring was more predictive than display rule perceptions for both surface acting and well/ill-being indicators.

### Theoretical Contributions

This study contributes to the literature of emotional labor by its focus on KBS workers and the unique characteristics of their emotional labor. First, by using criteria for both ill-being (anxiety, depression) and well-being (enthusiasm, contentment), this study investigated the effects of both ELS in a more comprehensive manner. Consistent with previous findings (Hülsheger and Schewe, 2011; Humphrey et al., 2015), surface acting was maladaptive to KBS workers’ well-being and was positively associated with anxiety and depression, and negatively associated with enthusiasm and contentment. The effects of deep acting were much more beneficial, in that significant and positive relationships were found between deep acting and both contentment and enthusiasm. Furthermore, the positive relationship between deep acting and anxiety and the non-significant one between deep acting and depression indicated that a moderate level of anxiety related to deep acting is evidence of dedication and engagement, rather than a proxy for psychological dysfunction (Humphrey et al., 2015).

Second, the finding that self-monitoring had stronger effects on participants’ use of ELS than display rule perceptions is consistent with previous theories. It seems that internalized values and rules are typically more predictive of individuals’ practices and behaviors than external demands or rules (Reeve et al., 2007). In other words, display rule perceptions are a more distal predictor and self-monitoring a more proximal predictor of individual behaviors regarding emotion regulation and management. In addition, there were stronger relationships between self-monitoring and surface acting and between display rule perceptions and deep acting. As mentioned earlier, previous studies have presented contradictory hypotheses and results concerning the effects of self-monitoring, and many have suggested that self-monitoring is inherently related to more efficient emotion regulation and adaptive strategies, such as deep acting (Bono and Vey, 2007; Scott et al., 2012). However, the present results indicate that for KBS workers, self-monitoring may not be as beneficial as previously thought. One plausible explanation is that self-monitoring only evokes or enhances the awareness of situational cues and the tendency to perform emotional labor, but does not guarantee coping efficiency or effectiveness. In other words, the effects of self-monitoring on the process of emotional labor may not be as self-evidently beneficial or detrimental as other relevant individual characteristics, such as emotional intelligence (the ability to perceive and regulate emotions efficiently) or positive and negative affect (the baseline or general affect/moods of individuals, which increases or decreases the level of emotion-rule dissonance) (Grandey, 2000; Totterdell and Holman, 2003; Hülsheger et al., 2013). Researchers have also proposed that high self-monitors can comfortably use surface acting without damaging the self, that low self-monitors may choose deep acting to follow the display rules and at the same time be themselves, and that high self-monitors are more fit for short-term service encounters and low self-monitors for long-term service relationships (Day et al., 2002; Holman, 2003; Scott et al., 2012). Somewhat consistent with these proposals, the present results suggest a stronger relationship between KBS workers’ self-monitoring and surface acting and a maladaptive effect of self-monitoring on their well-being.

Third, although display rule perceptions were not positively related to enthusiasm and contentment, the overall effects of display rule perceptions seem to be beneficial after controlling for the effects of self-monitoring. The results show that display rule perceptions have a stronger relationship with deep acting than with surface acting. Display rule perceptions were only weakly though positively related to anxiety and depression, while self-monitoring was positively related to anxiety and depression, and negatively to enthusiasm and contentment. These findings suggest that display rule perceptions have a more beneficial effect than self-monitoring. This suggestion is plausible, given that a moderate level of work strain is acceptable or even beneficial because it suggests not ill-being or dysfunction but rather engagement and dedication (Humphrey et al., 2015). Concepts such as person-job fit, trait-behavior congruence, and role identification have long been topics of importance in the field of emotional labor (Ashforth and Mael, 1998; Gosserand and Diefendorff, 2005; Bono and Vey, 2007; Humphrey et al., 2015). Researchers have also suggested that only when employees are able to challenge, but finally accept, their job roles and rules can they fully identify with and commit to their professions (Ashforth and Mael, 1998; Humphrey et al., 2015). A higher level of display rule perceptions, thus, may indicate that KBS workers identify with the often implicit emotional aspects of their work and faithfully choose the deep-acting strategy to fulfill these extra requirements. Nevertheless, no positive indirect relationship was found between display rule perception and contentment or enthusiasm. It is because the negative indirect effects on contentment and enthusiasm via surface acting and the positive indirect effects via deep acting balanced each other out. Thus, to further enhance the beneficial roles of display rule perceptions, it is desirable to strengthen the positive relationship between display rule perceptions and deep acting while weaken that between display rule perceptions and surface acting. This could be achieved by several ways. Specifically, it is necessary to promote KBS workers’ understanding of and commitment to the emotional aspects of their works. Extra effort is also need to familiarize them with different ELS, especially the deep-level solutions.

Finally, consistent with some previous studies, this study did not validate a two-dimension structure of positive and negative display rule perceptions, but adopted a single-dimension measure of display rule perceptions that combines displaying positive emotions and hiding negative ones (Gosserand and Diefendorff, 2005; Goldberg and Grandey, 2007). It is meaningful and feasible to distinguish between positive and negative emotions in research of emotional labor. For example, the Discrete Emotions Emotional Labor Scale (DEELS) developed by Glomb and Tews (2004) distinguishes between genuine, faked, and suppressed positive and negative emotional displays. The DEELS has been widely adopted and is proved to have good psychometrics qualities (Mahoney et al., 2011). Thus, while it is also theoretically important to distinguish between positive and negative rule perceptions, and to find their different implications for individuals’ strain and well-being, more effort is needed to develop a valid and reliable scale that links the theoretical conceptualizations and hypotheses with the empirical results.

### Practical Implications

The results of this study bring some implications for school practitioners and KBS providers at large. First, while teachers and other KBS workers should always be granted the job autonomy and flexibility they deserve, it is also important to raise their awareness about the emotional requirements and display rules of their jobs. The combination of autonomy and requirements may facilitate the identifying process, and thus enhance KBS workers’ commitment. That is, when fully identified with the emotional aspects of their professions, KBS workers may tend to perceive the emotional requirements as challenge rather than as threat or hindrance (Lazarus and Folkman, 1984; LePine et al., 2005). The faithful effort and the use of deep acting may still be resource-depleting, but, at the same time, rewarding and inspiring.

Second, for service employees in general, a major cause of their anxiety and work strain comes from limited job autonomy and strict display rules. The support received from managers and colleagues could be helpful. Managers and organizations have to balance between the interests of customers and employees. Sometimes, it is necessary for a manager to punish an employee to satisfy an angry customer. However, it is also important for him or her to show emotional understanding and to comfort and support the employee. In the educational contexts, school principals and other administrative staffs should assume their responsibilities to create a supportive and trustworthy school environment. Furthermore, to enhance parent involvement and promote the understanding and cooperation between teachers and parents may also help in reducing teachers’ anxiety and work strain.

Third, the results showed that high self-monitors may still tend to use surface acting strategies such as faking and hiding. This is probably caused by a limited knowledge of emotional labor and relevant strategies. Traditionally, the selection and training of KBS workers may focus on their professional competences. Limited support is provided to help them understand the emotional aspects of their jobs and use more adaptive strategies such as deep acting. Thus, programs aimed to improve employee mindfulness and emotional intelligence will be helpful for both teachers and KBS workers in general. Additionally, emotionally supportive environments could be useful. Platforms that allow for emotional communication and understanding may help KBS workers to recover from both relationship-related and profession-related strain at work.

### Limitations and Directions for Future Research

A major limitation of this study lies in its cross-sectional design and subjective data sources. While it is common to use self-reported data to measure personal perceptions and behavioral tendencies, sole reliance on self-reported data is vulnerable to social desirability bias and constrains the credibility of the results. Moreover, the use of cross-sectional design cannot exclude the possibility of reverse causality. Although it is theoretically convincing for display rule perceptions and self-monitoring to influence emotional labor and the consequential well-being of the participants (Grandey, 2000; Totterdell and Holman, 2003), it is possible that well-being may influence intrapersonal-level variations in self-monitoring tendencies or strategy adoption (Hülsheger et al., 2013). Further studies with longitudinal designs and multiple data sources can advance this line of research.

While this study has focused on KBS workers and used a sample of teachers, future studies are needed to confirm these relationships among other KBS occupations. With a particular focus on the effects of display rule perceptions, researchers may also compare the results of KBS workers and those of mass service employees. In addition, demographic factors (e.g., gender, age, tenure, education), characteristics of organizations (e.g., grade levels; private vs. public companies/schools; commercial vs. consumer-oriented law firms), and the cultural environments will definitely influence the ways in which KBS workers interact with their clients and perform their emotional labor. For example, professors at universities and teachers from primary and secondary schools play different roles in facilitating student learning. The interactions between professors and students are less frequent but any impropriate conducts of professors may lead to negative evaluations from students, which further influence their general evaluations and career developments. By contrast, school teachers, especially primary school teachers, may express their emotions and attitudes more frequently and directly (Hargreaves, 2000). The intensive emotional interactions between teachers and students also require more effort on the part of teachers. It is meaningful and important to test the potential moderating effects and compare group differences in future studies. It also would be worthwhile to test other criteria such as employee commitment or role identification and customer satisfaction or feedback (Hennig-Thurau et al., 2006; Groth et al., 2009).

## Conclusion

Based on the unique characteristics of KBS relationships, this study has investigated how self-monitoring and display rule perceptions are related to KBS workers’ ELS and the consequential well-being indicators of anxiety, depression, enthusiasm, and contentment. For KBS workers, who are generally less aware of the emotional aspects of their jobs and proper coping strategies, self-monitoring seems to be more related to a reactive emotional labor strategy (surface acting) than a proactive one (deep acting). By contrast, a higher level of display rule perceptions may represent role identification and commitment, and thus is related to a dedicated use of deep acting. Despite some limitations, this study illustrates characteristic aspects of emotional labor in KBS relationships and differentiates the roles of self-monitoring and display rule perceptions.

## Ethics Statement

Consistent with institutional review board procedures, this study was carried out in accordance with the recommendations of Survey and Behavioral Research Ethics Committee at the Chinese University of Hong Kong with written informed consent from all subjects. All subjects gave written informed consent in accordance with the Declaration of Helsinki. The protocol was approved by the Survey and Behavioral Research Ethics Committee.

## Author Contributions

SH analyzed the data and wrote the first version of the manuscript. HY designed the research and finished the final version of the manuscript. LT collected the data and helped with the data analysis.

## Conflict of Interest Statement

The authors declare that the research was conducted in the absence of any commercial or financial relationships that could be construed as a potential conflict of interest.
